# Characterization of the Proteomic Response in SIM-A9 Murine Microglia Following Canonical NLRP3 Inflammasome Activation

**DOI:** 10.3390/ijms27020689

**Published:** 2026-01-09

**Authors:** Nicolas N. Lafrenière, Karan Thakur, Gerard Agbayani, Melissa Hewitt, Klaudia Baumann, Jagdeep K. Sandhu, Arsalan S. Haqqani

**Affiliations:** 1 Department of Biochemistry, Microbiology and Immunology, University of Ottawa, Ottawa, ON K1H 8M5, Canada; nlafr060@uottawa.ca; 2 Human Health Therapeutics Research Centre, National Research Council Canada, Ottawa, ON K1A 0R6, Canada; kthak061@uottawa.ca (K.T.); melissa.hewitt@nrc-cnrc.gc.ca (M.H.); kbaum074@uottawa.ca (K.B.); 3 Department of Cellular and Molecular Medicine, University of Ottawa, Ottawa, ON K1H 8M5, Canada

**Keywords:** inflammasomes, innate immunity, neurodegenerative diseases, neuroinflammation, mass spectrometry, microglia, model systems, pyroptosis, Sample-Preparation by Easy Extraction and Digestion (SPEED), Single-Pot, Solid-Phase-Enhanced Sample-Preparation (SP3), Gel Enhanced Liquid Chromatography (GeLC)

## Abstract

Neuroinflammation is a hallmark of both acute and chronic neurodegenerative diseases and is driven, in part, by activated glial cells, including microglia. A key regulator of this inflammatory response is the NLRP3 inflammasome, an immune sensor that can be triggered by diverse, unrelated stimuli such as pathogen-associated molecular patterns, cellular stress, and mitochondrial dysfunction. Despite progress in targeting NLRP3-mediated immune activation, many drug candidates fail, potentially due to the limited availability of physiologically relevant disease models. The SIM-A9 murine microglial cell line, established in 2014, has emerged as a widely used model for studying neuroinflammation; however, its proteome has not yet been systematically characterized. In this study, we investigated the proteomic landscape of SIM-A9 microglia treated with classical pro-inflammatory stimuli, including lipopolysaccharide (LPS) and extracellular ATP and nigericin (NG), to induce NLRP3 inflammasome activation. Using complementary proteomic approaches, we quantified 4903 proteins and observed significant enrichment of proteins associated with immune and nervous system processes. Differentially expressed proteins were consistent with an activated microglial phenotype, including the upregulation of proteins involved in NLRP3 inflammasome signaling. To our knowledge, this is the first comprehensive proteomic analysis of SIM-A9 microglia. These findings provide a foundational resource that may enhance the interpretation and design of future studies using SIM-A9 cells as a model of neuroinflammation.

## 1. Introduction

Neuroinflammation is a hallmark of neurodegenerative diseases, including Alzheimer’s and Parkinson’s disease. A major contributor to this pro-inflammatory microenvironment is the activation of microglia, the brain’s resident immune cells, which exhibit phagocytic functions like peripheral macrophages [1]. Microglia possess a diverse repertoire of pattern recognition receptors (PRRs) that enable them to detect a wide array of danger cues, known as damage- or pathogen-associated molecular patterns (DAMPs and PAMPs). In the brain, these cues include extracellular adenosine triphosphate (ATP) from dying or dead cells, pathological protein aggregates such as α-synuclein, hyperphosphorylated tau and amyloid-β, heat shock proteins (HSPs), mitochondrial DNA (mtDNA), high-mobility group box 1 (HMGB1) protein, and pathogen-derived molecules such as lipopolysaccharide (LPS) or nigericin (NG). In microglia, these signals converge to activate the NLRP3 inflammasome pathway, primarily through nuclear factor kappa-B (NF-κB) signaling [2].

Activation of the canonical NLRP3 inflammasome is described as a two-step process. A priming stimulus (signal 1), acting through NF-κB, induces the transcriptional upregulation of the nucleotide-binding domain, leucine-rich repeat and pyrin domain-containing 3 (NLRP3), the adaptor protein ASC (apoptosis-associated speck-like protein containing a caspase recruitment domain), and the pro-forms of interleukin-1β (pro-IL-1β), and interleukin-18 (pro-IL-18). Despite this increase in transcript abundance, a second stimulus (signal 2) is required for protein-level upregulation and assembly of the mature NLRP3 inflammasome complex. Signal 2 promotes oligomerization of NLRP3 and ASC, as well as recruitment of caspase-1 and never in mitosis A-related kinase 7 (NEK7), enabling full inflammasome activation. The mechanistic details of the NLRP3 inflammasome assembly have been comprehensively reviewed elsewhere [3]. Once assembled, activated caspase-1 cleaves pro-IL-1β and pro-IL-18 into their mature, secreted forms, IL-1β and IL-18, respectively, and processes Gasdermin D (GSDMD) to form membrane pores that trigger pyroptosis, a lytic, inflammatory form of programmed cell death. Pyroptotic cells release cytokines, ATP, HSPs, mtDNA, and large DAMPs such as HMGB1, lactate dehydrogenase (LDH), and micrometer-sized extracellular ASC specks [4]. These molecules propagate inflammatory signaling in neighbouring innate immune cells, driving a feed-forward inflammatory cycle that underscores neuroinflammation as a critical therapeutic target in neurodegenerative diseases.

Primary human and rodent microglia are widely used to investigate microglial contributions to neurodegeneration and neuroinflammation, as well as to evaluate anti-inflammatory therapeutics [5,6]. Although primary cultures offer the highest physiological relevance, their use is limited by scarce availability, donor-to-donor variability, and ethical and logistical constraints. Further, they are labour-intensive, technically demanding, time-consuming, and costly. As a result, immortalized microglia cell lines, such as the murine BV2, generated using oncogenic transformation, is commonly used [7,8]. SIM-A9 is a more recent murine microglial cell line derived from a mixed glial culture of postnatal cerebral cortices that spontaneously immortalized, as reported by Nagamoto and Combs in 2014 [9]. It has since been widely employed to investigate microglia-mediated neuroinflammation, neurotrophic pain, and probe other specialized research questions [10,11]. Of important therapeutic relevance, SIM-A9 microglial cells have also been used to evaluate candidate anti-inflammatory compounds [12,13,14,15]. While transcriptomic and lipidomic characterizations of SIM-A9 microglia have been reported, no untargeted proteomic dataset currently exists to provide a complementary intermediary between these omics technologies [16,17].

In this study, we aimed to characterize proteome-wide changes in parental SIM-A9 cells following stimulation with classical pro-inflammatory stimuli, LPS and the NLRP3 inflammasome inducers ATP and nigericin (NG). In parallel, we evaluated several commonly used proteomic sample-preparation methods to determine how methodological choices influence proteomic coverage and the detection of inflammation-associated signatures.

## 2. Results

### 2.1. Characterization of Inflammatory Responses in SIM-A9 Microglia

**Figure 1:**
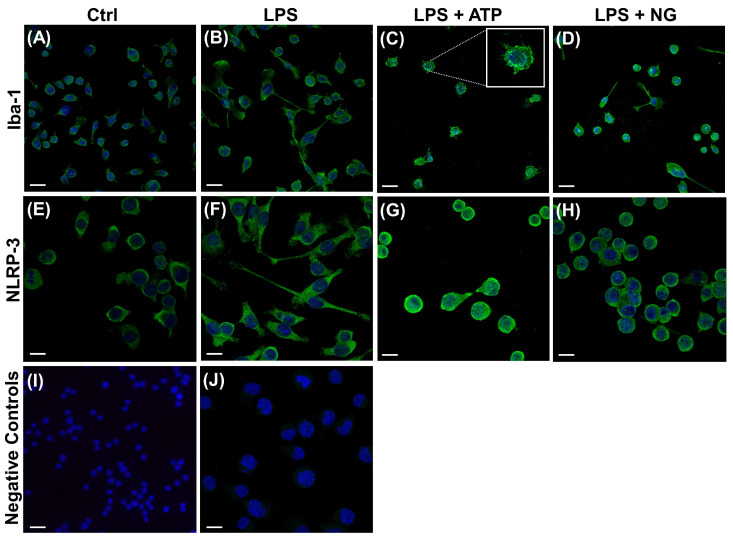
Immunofluorescence analysis of SIM-A9 microglia. SIM-A9 cells were treated in serum-free medium with LPS (3 h), followed by stimulation with ATP or NG (1 h). Controls were maintained in serum-free medium for 4 h, as described in Methods. Panels show: (**A**,**E**) Untreated controls (Ctrl); (**B**,**F**) LPS-primed cells (LPS); (**C**,**G**) LPS + ATP; and (**D**,**H**) LPS + NG. Cells were stained with anti-Iba-1 (**A**–**D**) or NLRP3 (**E**–**H**) antibodies. The inset in panel (**C**) highlights amoeboid-shaped microglia. Iba-1 or NLRP3 immunoreactivity is shown in green, and nuclei counterstained with NucBlue are shown in blue. Panels (**I**,**J**) show negative controls in which cells were incubated with goat anti-rabbit IgG-Alexa 488 and goat anti-mouse IgG-Alexa 488, respectively. Images are representative of three independent experiments. Scale bars: 75 µm (**A**–**D**,**I**) and 50 µm (**E**–**J**).

SIM-A9 cells grew as an adherent monolayer and exhibited heterogeneous, microglia-like morphologies. Under phase-contrast microscopy, most cells appeared flattened and polygonal, while a subset displayed bipolar shapes or extended multiple processes. To confirm microglial identity, we performed immunofluorescence staining for markers previously reported in SIM-A9 cells [9]. As expected, cells were positively immunostained for ionized calcium binding adaptor molecule-1 (Iba-1, also known as AIF1; Figure 1A), a microglia marker known to be modulated by inflammatory signals [18]. SIM-A9 microglia also expressed CD68, a microglia/macrophage marker (Supplementary Figure S1). No astrocytic or neuronal differentiation was detected, as indicated by the absence of glial fibrillary acidic protein (GFAP) or microtubule-associated protein 2 (MAP2) immunoreactivity (Supplementary Figure S1), consistent with the previous report [9].

We next examined whether inflammatory stimulation altered Iba-1 expression or cell morphology. SIM-A9 cells were primed with LPS for 3 h and subsequently stimulated for 1 h with canonical NLRP3 inflammasome activators ATP, which signals through the P2X7 purinergic receptor, or NG, which acts as a potassium ionophore and induces K+ efflux (Figure 1B–D). LPS priming alone induced a modest increase in bipolar morphology and Iba-1 expression (Figure 1B). In contrast, ATP or NG treatment caused marked morphological changes, with cells transitioning from polygonal to rounded and amoeboid shapes accompanied by increased Iba-1 expression, features consistent with microglial activation (Figure 1C,D).

The treatment protocol used in these studies was informed by pilot experiments employing real-time cell analysis with the IncuCyte system (Supplementary Figure S2). In cultures treated with ATP or NG, a steady increase in SYTOX Green-positive cells and a reduction in cell numbers was observed, indicating microglia underwent cell death (Supplementary Figure S2A–C). Quantification of cell death revealed that cell loss was minimal at 4 h (panel C), increased significantly by 12 h, and by 22 h almost all cells were dead (panel B). At the 22-h time point, both untreated SF (Ctrl) cultures and LPS-primed cultures continued to proliferate and showed no increase in SYTOX Green-positive cells (panels A and C). Based on these phenotypic changes in SIM-A9 microglia, we next investigated whether these stimuli triggered molecular markers of NLRP3 activation.

Treatment of mouse microglial cells with LPS followed by inflammasome inducers is known to upregulate NLRP3 inflammasome components [19]. To determine whether SIM-A9 microglial cells exhibit a similar response, we performed immunofluorescence staining using an anti-NLRP3 antibody (Figure 1E–H). In untreated SIM-A9 cells, NLRP3 exhibited diffuse cytoplasmic expression (Figure 1E) and LPS priming increased overall NLRP3 levels, although the protein remained evenly distributed throughout the cytoplasm (Figure 1F). In contrast, cells treated with LPS followed by ATP or NG showed more pronounced and localized NLRP3 protein enrichment at the plasma membrane (Figure 1G,H). Together, these findings demonstrate that SIM-A9 microglia mounts a robust NLRP3 inflammasome response following ATP or NG stimulation.

**Figure 2:**
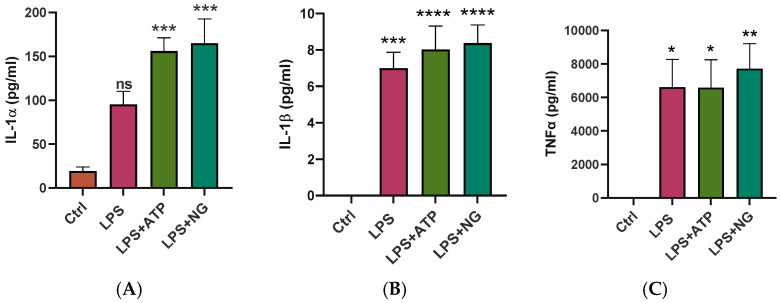
Measurement of pro-inflammatory cytokine release in SIM-A9 cells following exposure to canonical NLRP3 inflammasome stimuli. SIM-A9 cells were primed in serum-free (SF) medium with LPS for 3 h, followed by stimulation with ATP or NG for 1 h. Controls were maintained in SF medium for 4 h, as described in the Methods Section. Following treatments, levels of IL-1α (**A**), IL-1β (**B**) and TNFα (**C**) secreted into the culture supernatants were measured using a multiplex bead-based immunoassay. Data are presented as mean ± SEM from two independent experiments. In each experiment, SIM-A9 cells were treated in duplicate wells of a 24-well plate, and supernatants from each well were analyzed in duplicate. Statistical analysis was performed using one-way ANOVA followed by Tukey’s multiple comparisons test. Statistically significant differences between treatment groups (LPS, LPS + ATP and LPS + NG) compared with the SF control are indicated as * *p* < 0.05, ** *p* < 0.01, *** *p* < 0.001, and **** *p* < 0.0001. Differences were considered non-significant (ns) with *p* > 0.05.

We next examined whether SIM-A9 microglia respond to inflammatory stimulation by increasing cytokine production. After 4 h of incubation in serum-free medium (Ctrl) detectable amounts of IL-1α (19.5 pg/mL) were present in the culture supernatants, whereas IL-1β and TNFα were not detected (Figure 2). Treatment with LPS induced significant production of IL-1α, IL-1β and TNFα, reaching concentrations of 95.32 ± 14.95 pg/mL (Figure 2A), 7.0 ± 0.87 pg/mL (Figure 2B) and 6614 ± 1653 pg/mL (Figure 2C), respectively. LPS priming followed by stimulation with ATP or NG further enhanced cytokine release. As compared to untreated SF controls, IL-1α and IL-1β levels increased ~8-fold in LPS + ATP and LPS + NG-treated cultures (Figure 2A,B), while TNFα levels showed a robust increase of more than 6000-fold relative to SF controls (Figure 2C).

IL-1α acts as an early danger signal (alarmin) released upon cell stress or membrane damage, whereas IL-1β is a canonical inflammasome-dependent cytokine that indicates activation of the NLRP3 pathway in microglia. Both IL-1α and IL-1β bind to the IL-1 receptor and exert overlapping proinflammatory effects. TNFα is a rapid-response cytokine that promotes inflammation, immune cell recruitment, and amplification of the cytokine cascade. Together, these results demonstrate that SIM-A9 microglia are functionally active and mount an appropriate pro-inflammatory response to stimulation.

### 2.2. The Proteome of SIM-A9 Microglia

**Figure 3:**
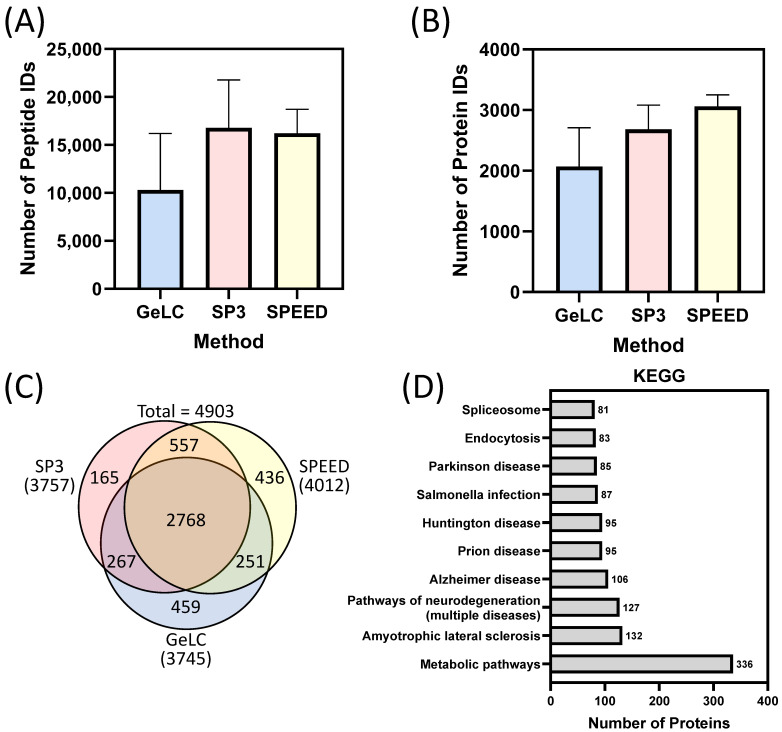
(**A**) Number of peptide and (**B**) protein IDs using the GeLC, SP3, SPEED sample-preparation methods (*n* = 3). (**C**) Venn diagram of protein IDs identified using all methods, SP3, SPEED and GeLC, proteins needed to be confidently identified in at least 1 of 3 replicates. (**D**) All 4903 proteins from (**C**) were subjected to enrichment analysis using the Kyoto Encyclopedia of Genes and Genomes (KEGG) disease database; the 10 terms with the greatest intersection with the query terms were graphed. The data are presented as mean ± SEM where applicable.

As SIM-A9 cells were shown to exhibit characteristic microglia-like morphology and activate the NLRP3 inflammasome, we next conducted a comprehensive proteomic analysis. To maximize proteome coverage, we used three complementary sample-preparation methods: (1) SP3 (Single-Pot, Solid-Phase-enhanced Sample-Preparation), (2) SPEED (Sample-Preparation by Easy Extraction and Digestion), two rapid in-solution protocols [20,21], and (3) GeLC, (Gel Enhanced Liquid Chromatography) an in-gel digestion approach that enhances the detection of higher-molecular-weight or lower-abundance proteins. Mass spectrometry raw data files were batch-processed according to sample-preparation method to avoid matching-between-runs for proteins identified by different workflows. Across all methods, we identified 4903 proteins in SIM-A9 cells. SP3 and GeLC produced comparable proteome depths (3757 and 3745 proteins, respectively) (Figure 3A,B), whereas SPEED yielded the highest number of identified proteins (4012).

Each method contributed a distinct set of proteins to the overall proteome. GeLC identified 459 proteins not detected by SP3 or SPEED, while SP3 and SPEED identified 165 and 436 proteins, respectively (Figure 3C). As expected, GeLC preferentially captured higher-molecular-weight proteins compared with the in-solution methods (SP3 and SPEED; Supplementary Figure S3A,B). In contrast, SPEED favored smaller proteins: 146 proteins <20 kDa, compared with 50 by SP3 and only 2 by GeLC. A similar trend was observed for proteins in the 20–40 kDa range (134 proteins by SPEED, 91 proteins by SP3, and 80 proteins by GeLC).

Differences extended beyond protein size. SP3 tended to identify more basic proteins, reflected by a higher median isoelectric point (pI) (Supplementary Figure S3), whereas SPEED enriched acidic proteins with pI values between 4 and 5, consistent with its underlying chemistry. Membrane topology further distinguished the methods: SP3 consistently recovered more transmembrane domain (TMD)-containing proteins, including many with multiple TMDs (4, 7, or 12). Taken together, these complementary biases demonstrate that integrating GeLC, SP3, and SPEED provides broader proteome coverage across molecular weight, charge, and membrane classes than any single method alone.

A substantial portion of the proteome, 2768 proteins, was shared across all three preparation methods (Figure 3C). Within this common protein set, at least 100 proteins mapped to KEGG (Kyoto Encyclopedia of Genes and Genomes) pathways associated with neurodegenerative and inflammatory diseases, including Alzheimer’s, Parkinson’s, and Huntington’s diseases, as well as Salmonella infection (Figure 3D) [22]. These pathways are highly relevant to microglial function, as microglia are central mediators of neuroinflammation and are directly implicated in the progression of these diseases through roles in cytokine production, phagocytosis, synaptic surveillance, and inflammasome activation [23,24]. As expected for a whole-cell proteome, core metabolic pathways were among the most enriched KEGG categories. This broad metabolic representation aligns with diverse bioenergetic and immunometabolic programs that support microglial functions in surveillance, activation, and response to injury or infection. Additional overrepresentation analyses using G:Profiler, version e113_eg59_p19_f6a03c19, database updated on 23 May 2025 (Supplementary Table S1) further reinforced these themes and were consistent with the expected landscape of whole-cell microglial cell lysates.

To further validate the microglial origin of SIM-A9 cells, we examined their proteome for established brain cell-type specific markers. A curated set of more than 500 markers representing major brain cell populations, including microglia, oligodendrocytes, astrocytes, endothelial cells, and neurons compiled from transcriptomic datasets [25,26] was mapped against the SIM-A9 proteome. This analysis revealed strong enrichment of microglial lineage-defining proteins. We confidently identified 87 microglia-enriched proteins in SIM-A9 cells (Supplementary Table S2), including canonical markers such as Aif1/Iba1, Csf1r, CD68, C1qb, C1qc, and Pld4. These proteins participate in essential microglial processes: Csf1r mediates survival, proliferation, and homeostatic maintenance. Aif1/Iba1 regulates calcium-dependent actin remodeling and motility facilitating rapid surveillance. Complement components C1qb and C1qc act within the complement cascade for synaptic pruning, and CD68 participates in phagolysosomal trafficking and lysosomal degradation [27]. In contrast, markers specific to neurons, astrocytes, oligodendrocytes, and endothelial cells were largely absent in our dataset, reinforcing that SIM-A9 cultures maintain a predominantly microglial molecular identity.

**Figure 4:**
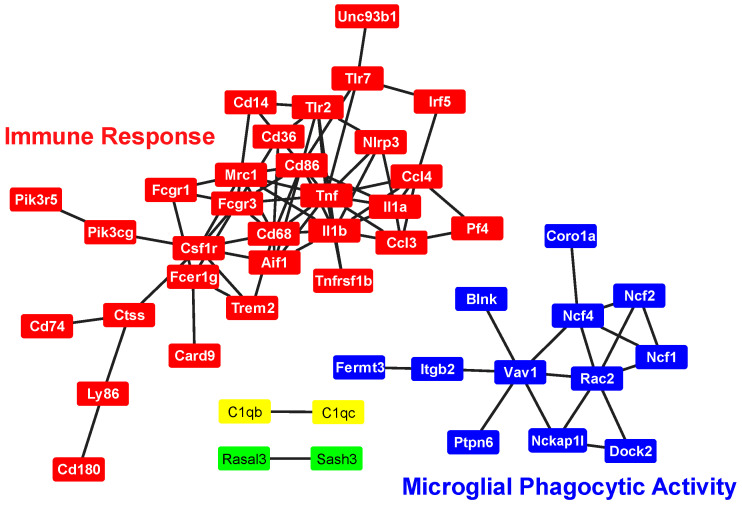
STRING Protein–protein interaction map. All 87 microglial markers identified in the SIM-A9 proteome were analyzed using the STRING database, and only interactions with a score >0.8 are shown. Four distinct interaction networks were identified, two small clusters of 2 proteins each, a medium-sized cluster of 12 proteins, and a large cluster comprising 30 proteins. Edge colors correspond to the type of evidence supporting each interaction.

STRING protein–protein interaction analysis revealed that the 87 microglial markers organize into two mechanistically coherent modules (Figure 4). The largest cluster (30 proteins) was enriched for innate immune signaling nodes, including high-degree hubs such as CD68 and CD86, which interface with pattern-recognition pathways (e.g., TLR–MyD88 and C-type lectin receptors) to regulate phagosome maturation, antigen presentation and NF-κB activation and transcription of pro-IL-1β, and priming of the NLRP3 inflammasome [24]. This cluster also included complement components, scavenger receptors, and chemokine regulators, highlighting functional pathways involved in debris clearance, microbial sensing, cytokine production, and recruitment of peripheral immune cells.

The second major cluster (12 proteins), anchored by Rac2 and Vav1, mapped to cytoskeletal remodeling and phagocytic engulfment machinery. Rac2-Vav1 signaling drives assembly of the phagocytic cup, membrane ruffling, and oxidative burst responses [28], linking SIM-A9 to the canonical microglial pathways required for synapse elimination, apoptotic cell clearance, and pathogen removal [29]. Together, these mechanistic networks demonstrate that SIM-A9 cells express an integrated microglial program encompassing survival signaling, innate immune activation, complement-mediated synaptic remodeling, and actin-driven phagocytosis. These features strongly support the microglial identity of SIM-A9 cells and highlight their relevance as a model for studying microglial signaling pathways.

### 2.3. Response of SIM-A9 Microglia to Classical Pro-Inflammatory Stimuli

**Figure 5:**
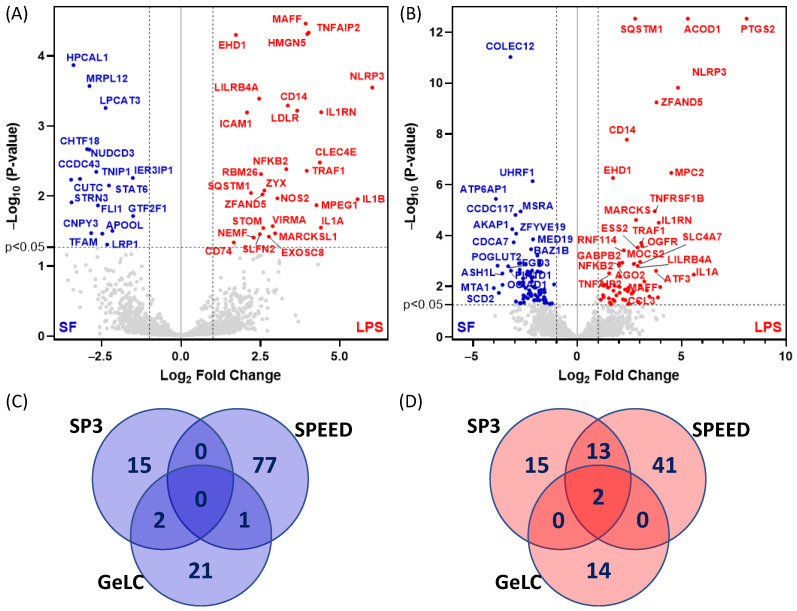
Volcano plot analysis of proteins differentially expressed in response to LPS priming using (**A**) SP3 and (**B**) SPEED proteomic workflows. Venn diagram analysis of commonly identified dysregulated proteins using both proteomic methods: (**C**) proteins with decreased expression and (**D**) proteins with increased expression following LPS priming. In panels (**A**,**B**), the vertical dashed lines correspond to 2-fold change (i.e., Log_2_ of either −1 or 1), whereas the horizontal dashed line correspond to *p* value < 0.05.

We next investigated the proteomic response of SIM-A9 cells primed with LPS and subsequently treated with ATP or NG, as described in Section 4.1. Proteins were analyzed using GeLC, SP3, and SPEED workflows. Differentially expressed proteins were identified as those with a statistical significance *p*-value < 0.05 (One-way ANOVA followed by Tukey test) and a >1.5-fold change relative to untreated serum-free (SF) controls. Figure 5A,B illustrate the changes in protein expression following LPS stimulation. Using the SP3 method, LPS treatment significantly altered the expression of 47 proteins (30 upregulated, 17 downregulated), whereas SPEED detected 134 differentially expressed proteins (56 upregulated, 78 downregulated), indicating that SPEED captured a broader spectrum of proteomic changes (Figure 5A,B). Importantly, 15 proteins were consistently upregulated across both methods (Figure 5D), while no proteins were significantly downregulated across both methods (Figure 5C), revealing a convergent upregulation signature in response to LPS. These commonly upregulated proteins include well-known LPS-responsive inflammatory markers, such as NLRP3 (the inflammasome sensor), PTGS2 (Prostaglandin G/H synthase 2, also known as COX-2), and TNFRSF1B (tumor necrosis factor receptor type II). For NLRP3, multiple unique peptides showed increased intensity in LPS-treated cells, corresponding to an approximately 2–3-fold increase in protein abundance relative to untreated controls in our data, consistent with robust inflammasome priming.

**Figure 6:**
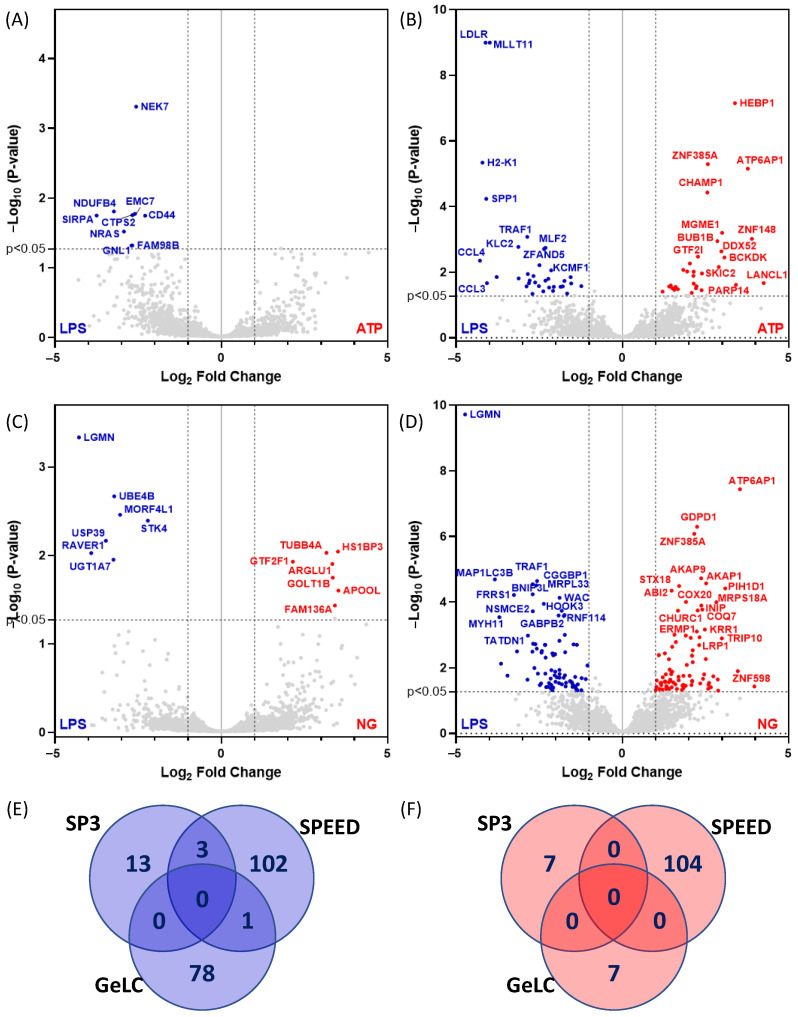
Volcano plot analysis of ATP-treated samples relative to LPS-treated samples using (**A**) SP3 and (**B**) SPEED, and NG-treated samples relative to LPS-primed samples using (**C**) SP3 and (**D**) SPEED proteomic workflows. Significance was defined as *p* value < 0.05 and log2 fold change ≥ 1.5. As ATP and NG were applied following LPS priming, their effects were assessed relative to LPS. (**E**,**F**) Venn diagram analysis of proteins showing down-regulation (**E**) or up-regulation (**F**) in response to ATP and NG across SP3, GeLC, and SPEED workflows, highlighting key regulators of inflammasome activation and secondary signaling. In panels (**A**–**D**), the vertical dashed lines correspond to 2-fold change (i.e., Log2 of either −1 or 1), whereas the horizontal dashed line correspond to *p* value < 0.05.

As NLRP3 was identified as an upregulated protein in the proteomic analysis, we next tested the effects of adding a secondary stimulus to promote full NLRP3 inflammasome activation. All treatments were carried out in SF conditions; SIM-A9 microglia were primed with LPS and then treated with inflammasome inducers. We selected ATP, which models sterile inflammation and NG, a potassium ionophore that induces K + efflux, a key trigger of inflammasome assembly. As ATP and NG were added after LPS priming, LPS-treated cells without a second signal served as the reference condition for these comparisons (Figure 6). Distinct differential protein expression patterns were observed in each treatment group (Figure 6), with the SPEED method identifying a larger number of differentially expressed proteins (Figure 6E).

The differentially observed proteins following LPS, LPS + ATP, and LPS + NG treatments were further examined using functional enrichment (GO/KEGG/Reactome) to identify if any specific processes and pathways are differentially regulated (Supplementary Table S3). The proteins mapped very strongly to a coherent innate immune/inflammasome-driven activation program, with enrichment across GO/KEGG/Reactome for NLRP3/inflammasome-related processes, broad cytokine and chemokine signaling, and TLR-MyD88-NFkappaB/TNF-axis inflammatory signaling, consistent with canonical priming (LPS) plus secondary activation (ATP and/or NG) driving IL-1 family outputs. In addition, the proteins also mapped to an oxidative/nitrosative stress and immune–metabolic response (e.g., inducible nitric oxide synthase (Nos2) plus redox/defense-associated factors like oxidation resistance 1 (Oxr1), and heme oxygenase 2 (Hmox2)).

**Figure 7:**
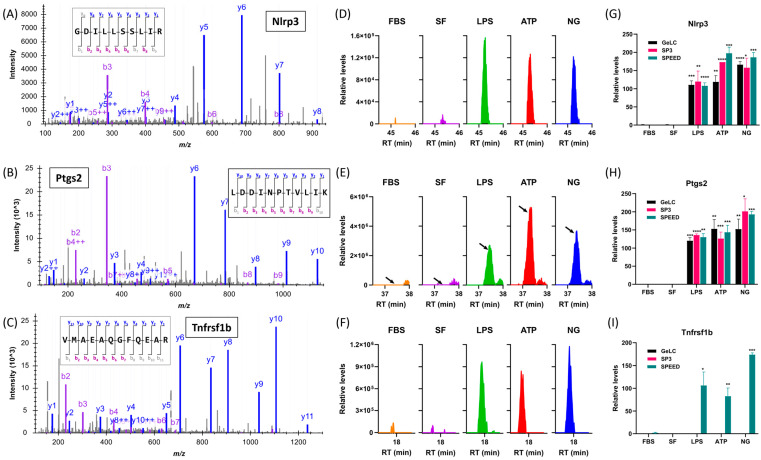
Examples of microglial proteins showing differential expression in response to treatment. Shown are representative MS/MS spectrum (**A**–**C**), representative extracted ion chromatogram (**D**–**F**) and overall relative change (**G**–**I**) for Nlrp3 (**A**,**D**,**G**), Ptgs2 (**B**,**E**,**H**) and Tnfrsf1b (**C**,**F**,**I**). The spectra correspond to precursor peptides GDILLSSLIR (*m*/*z* 543.8295, charge 2+, RT 45.3 min) for Nlrp3, LDDINPTVLIK (*m*/*z* 620.8610, charge 2+, RT 37.3 min) for Ptgs2, and VMAEAQGFQEAR (*m*/*z* 668.8193, charge 2+, RT 17.8 min) for Tnfrsf1b. Each peptide had PepProphet probability ≥0.99, corresponding to a 1% FDR at the PSM level. These proteins are key regulators of inflammasome activation and inflammatory signaling, confirming the proteomic changes observed in response to LPS or secondary stimuli (ANOVA followed by Tukey’s test showing *p* < 0.05 (*), *p* < 0.01 (**), *p* < 0.001 (***) and *p* < 0.0001 (****) relative to SF controls in (**G**–**I**). In panel (**G**), arrows indicate the peak that corresponds to the Ptgs2 peptide. Note that in panel I, Tnfrsf1b was only detected by SPEED. Skyline (64-bit) 25.1.0.237 (519d29babc) was used for MS data plotting in panels (**A**–**F**) and GraphPad Prism version 10.6.1 (64-bit, 892) was used for results plotting and statistical analysis in panels (**G**–**I**).

Finally, to validate the proteomic findings, we manually inspected MS/MS spectra for microglial markers identified as modulated in response to pro-inflammatory stimulation. Representative spectra are shown in Figure 7, confirming the identification of NLRP3, PTGS2 (COX-2) and TNFRSF1B in stimulated samples. Consistent with the quantitative proteomic data, peptide signal intensities for each protein were higher in LPS- and LPS plus ATP/NG-treated conditions compared to unstimulated controls, verifying their induction at the protein level. These proteins are key regulators of inflammasome activation and inflammatory signaling, supporting the functional relevance of the observed proteomic changes.

## 3. Discussion

In this study, we present a comprehensive proteomic characterization of the SIM-A9 murine microglial cell line, combining three complementary sample-preparation workflows to maximize protein coverage. With over 4900 proteins identified, this dataset represents one of the most extensive proteomic profiles reported for an immortalized microglial model, providing a valuable reference for the field. The proteome was highly enriched for microglia-specific proteins, with minimal detection of markers from other brain cell types, such as neurons, astrocytes or endothelial cells, confirming the fidelity of SIM-A9 as a representative microglial model.

Functionally, SIM-A9 cells mounted a robust inflammatory response to classical NLRP3 inflammasome stimuli. LPS priming followed by ATP or NG treatment led to increased abundance of key inflammatory mediators, including NLRP3, PTGS2 (COX-2), and TNFRSF1B, as validated by manual inspection of MS/MS spectra. These findings confirm that SIM-A9 cells retain core microglial functions, including inflammasome activation and downstream inflammatory signaling, highlighting their suitability for mechanistic studies of microglial-driven neuroinflammation. Importantly, our multi-method proteomic approach enabled detection of both high- and low-abundance proteins, revealing a broad and functionally relevant protein landscape that might be overlooked using single-method workflows. By providing a detailed proteomic reference for SIM-A9 cells under both basal and stimulated conditions, this work offers a foundation for future studies investigating microglial activation pathways, inflammatory mechanisms, and potential therapeutic targets in neurodegenerative diseases.

### 3.1. SIM-A9 as a Microglial Model

Our data provide proteomic and immunocytochemical evidence supporting SIM-A9 as a valid microglial model system. The presence of microglial signature markers, including Iba-1 and CD68, together with microglia-like morphology, confirms that SIM-A9 cells closely resemble primary microglia in identity. Proteomic analysis revealed strong expression of numerous microglial-enriched proteins, such as C1qa, Trem2, and Csfr1, with minimal detection of neuronal or astrocytic markers (e.g., MAP2, GFAP), underscoring the purity and lineage specificity of the SIM-A9 cultures. Functionally, SIM-A9 cells respond to pro-inflammatory stimuli in a manner consistent with activated microglia. Upregulation of COX-2, NLRP3, and TNF pathway components mirrors the classical pro-inflammatory activation profile observed in primary microglia and macrophages following LPS and exposure to “danger” signals. These findings are in line with previous reports that SIM-A9 cells respond appropriately to pro- and anti-inflammatory agents, performing canonical microglial functions, such as cytokine secretion and phagocytosis of debris or pathogens [9]. Notably, SIM-A9 cells robustly induced NLRP3 inflammasome-associated proteins and downstream IL-1β release (Figure 2), demonstrating the preservation of this critical innate immune pathway. The LPS + ATP/NG two-signal paradigm used here is well-established for activating the NLRP3 inflammasome in microglia and has been successfully applied in other microglial cell lines to evaluate inflammasome inhibitors [30]. Collectively, our results reinforce that SIM-A9 cells share core phenotypic and reactive properties with primary microglia, supporting their utility as an in vitro model of neuroinflammation.

### 3.2. Comparison to Other Microglial Cell Lines

In addition to the SIM-A9 model, two commonly used microglial cell models include the murine BV2 line and the human hESC-derived microglia. BV2 cells, an older v-myc/v-raf immortalized line, have been extensively benchmarked against primary microglia. While they generally exhibit similar trends in activation, their responses are often blunted relative to primary cells. In contrast, SIM-A9 cells, derived via spontaneous immortalization, have been shown to respond to stimuli with potency comparable to primary microglia [9].

Proteomic comparisons have revealed substantial overlap between murine SIM-A9 and BV2, and the human hESC-derived microglial cell lines. Approximately 90% of the murine SIM-A9 proteome overlapped with the murine BV2 proteome, and about 85% overlapped with the human hESC-derived microglia proteome. This extensive overlap included 71 of the 87 microglia-enriched proteins identified in SIM-A9, indicating a shared core of microglial characteristics among these models. Notably, SIM-A9 also uniquely expressed a subset of important glial markers that have not been detected in either the BV2 or hESC proteomes. These included Nlrp3 (inflammasome sensor), Fcγ receptors Fcgr1 (CD64) and Fcgr3 (CD16), Clec10a (C-type lectin receptor), Slc2a5 (GLUT5 glucose transporter), Lyz2 (lysosomal enzyme), Ly86 (innate immune regulator MD-1), Tnfrsf1b (TNF receptor 2), Pf4 (platelet factor 4), Slfn2, Snx20 (sorting nexin 20), and several key inflammatory cytokines andchemokines (Il1a, Il1b, Tnf, Ccl3, Ccl4). The presence of these markers suggests that SIM-A9 retains a broad repertoire of innate immune and inflammatory responses. For example, Nlrp3 and its downstream effectors IL-1α, IL-1β, and TNFα are central to inflammasome-driven neuroinflammation, indicating that SIM-A9 can mount robust inflammasome responses, whereas other cell lines may be limited. Similarly, high-affinity Fc receptors (CD64, CD16) and TNFR2 expression in SIM-A9 support antibody-mediated phagocytosis and TNF signaling, functions that are characteristic of primary microglia. In summary, due to these unique features, the SIM-A9 microglia model provides a useful in vitro system for investigating mechanisms of inflammatory activation and innate immune function. Its molecular and functional profile offers researchers a consistent and physiologically relevant platform for exploring microglial responses, while complementing other established models such as BV2 and hESC-derived microglia.

### 3.3. Utility of Multi-Method Proteomics

A major strength of our study was the integration of three complementary proteomic workflows, which allowed us to overcome biases inherent to any single sample-preparation method. Different lysis and digestion techniques are known to favour specific subsets of the proteome [31]. In our experimental paradigm, the SPEED protocol, which employs trifluoroacetic acid, a strong acid for protein extraction, enhances the identification of small (<15 kDa) and acidic proteins that can be under-represented in conventional workflows. The harsh acid lysis used in the SPEED workflow likely solubilized proteins that are resistant to detergent-based methods. The SP3 method, employing detergent-assisted lysis (i.e., sodium deoxycholate, SDS) and magnetic bead capture, improved the recovery of hydrophobic, membrane-associated proteins in our study, as reflected by a higher proportion of identified transmembrane proteins in the SP3 dataset. GeLC, by contrast, provided an orthogonal separation based on molecular weight, enabling detection of very high-mass proteins and distinct proteoforms that might be missed in one-pot digests. GeLC enriches proteins with extreme size or domain composition, particularly in the upper molecular weight range.

Each workflow therefore “biased” protein detection toward specific physiochemical protein classes, yielding a set of unique protein identifications while maintaining substantial overlap; most proteins were detected by at least two methods. Importantly, the combination of these approaches incrementally increased overall proteome coverage. This multi-method strategy proved highly effective for comprehensive proteomic profiling, capturing a spectrum of proteins from small acidic cytokines to large membrane receptors. Such depth is particularly valuable for SIM-A9 cells, as it allows confident verification of microglial identity and NLRP3 inflammasome-associated pathways through lineage markers and enables broad exploratory analysis of treatment-induced changes across thousands of proteins.

We should also note, however, that the proteomics profiling was performed using DDA method, and over the recent years, data-independent acquisition (DIA) now represents the state-of-the-art method for both proteome depth and quantitative consistency. Future studies using the SIM-A9 model will incorporate DIA-based workflows, particularly for more sophisticated quantitative and pathway-level analyses of microglial and inflammasome biology.

### 3.4. Therapeutic and Research Implications

Our study demonstrates that SIM-A9 cells respond to inflammatory triggers with proteomic changes consistent with activated microglia, supporting their use as an in vitro platform for neuroinflammation research and therapeutic screening. The robust NLRP3 inflammasome response, upregulation of NLRP3 and IL-1β following LPS + ATP/NG stimulation, highlights their relevance for studies of inflammasome-targeting drugs in neurodegenerative diseases. Increasing evidence indicates that excessive microglial inflammasome activation contributes to neurodegeneration, and the NLRP3 inflammasome inhibition is being explored as a therapeutic strategy in conditions like Alzheimer’s and Parkinson’s disease [9,32,33,34].

The comprehensive proteomic dataset generated here under unstimulated conditions provides a baseline against which drug effects can be assessed. For example, compounds that attenuate SIM-A9 inflammatory responses would be expected to reduce levels of key mediators, including NLRP3, COX-2, or downstream cytokines. High-content proteomics also enables identification of off-target effects or pathway-specific signatures, offering insights into drug mechanisms of action. Furthermore, the immortalized, ease-of-culture nature of SIM-A9 cells facilitates high-throughput screening approaches that are challenging with primary microglia. High-throughput and high-content multi-well drug screening assays can be used to evaluate libraries of anti-inflammatory or neuroprotective compounds, measuring both secreted factors (e.g., cytokines) and intracellular proteomic changes. SIM-A9 are particularly suitable for testing NLRP3 inhibitors: small-molecular weight compounds, such as MCC950, novel therapeutic antibodies, or other modulators of inflammasome signaling [33,34].

In conclusion, deep, multi-method proteomic profiling confirms that SIM-A9 cells retain a core microglial identity and functional responses, offering an extensive dataset spanning thousands of proteins. While limitations of immortalized models should be acknowledged, the reproducibility, homogeneity, scalability, and broad proteome coverage of SIM-A9 microglia make them a powerful platform for mechanistic studies; particularly, its functional NLRP3 inflammasome activation could provide insights into inflammatory pathways and early-stage therapeutic screening.

## 4. Materials and Methods

### 4.1. Cell Culture

Murine SIM-A9 microglia (ATCC, Manassas, VA, USA; Cat# CRL-3265) were cultured in Dulbecco’s modified Eagle’s medium/Ham’s F12 50/50 Mix (DMEM/F12; Wisent, Poland, Belarus; Cat# 319-075-CL) supplemented with 10% heat-inactivated fetal bovine serum (FBS; Gibco, Waltham, MA, USA; Cat# 12483-020). This formulation is referred to as a complete medium. Cells were maintained under standard conditions at 37 °C and 5% CO_2_, and then passaged every 2–3 days as required in T25 flasks.

For proteomic experiments, 2.2 × 10^5^ SIM-A9 cells were seeded into each well of a 6-well plate in complete medium and incubated overnight. Untreated cells maintained in complete medium (10% FBS) served as controls. All treatments with inflammasome inducers were carried out in serum-free (SF) medium, lacking FBS supplementation. To prime the NLRP3 inflammasome, cells were treated with LPS (100 ng/mL) for 3 h. Following priming, inflammasome activation was induced by adding either 5 mM ATP or 5 µM NG for 1 h. For the LPS-only condition, cells were incubated for a total of 4 h. SF controls were maintained for 4 h under identical conditions. After treatment, the plate was centrifuged at 1000 rpm for 5 min; cells were scraped in 1 mL PBS, centrifuged at 1000 rpm for 5 min, and the resulting pellets were stored at −80 °C for mass spectrometry analysis. All experiments were conducted with a minimum of three biological replicates, and each sample was analyzed in duplicate.

### 4.2. Immunofluorescence Microscopy

SIM-A9 cells were seeded at a density of 5 × 10^4^ cells per well onto poly-L-lysine-coated glass coverslips placed into a 24-well plate. After overnight incubation, SIM-A9 cells were pre-treated with LPS and subsequently stimulated with the inflammasome inducers, ATP or NG, as described in Section 4.1. Following treatments, cells were rinsed with PBS and fixed with 4% paraformaldehyde for 20 min. For immunofluorescence staining, fixed cells were rinsed with PBS and permeabilized with 0.25% Triton X-100 for 10 min. Non-specific binding was blocked by incubating the cells with a serum-free protein block (Agilent, Santa Clara, CA, USA; Cat# X0909) for 20 min at room temperature. After removing excess block, cells were incubated overnight at 4 °C in a humidified chamber with anti-Iba-1 (1:1000, Fujifilm Wako, Osaka, Japan; Cat# 019-19741) or anti-NLRP3 (clone Cryo-2, 1:100, Adipogen, San Diego, CA, USA; Cat# AG-20B-0014-C100) antibodies, diluted in Dako antibody diluent (Agilent Dako). Cells were then rinsed twice with PBS and incubated for 45 min with goat anti-rabbit Alexa 488 or anti-mouse 488 secondary antibodies (1:500, Invitrogen, Carlsbad, CA, USA) diluted in antibody diluent. Omission of the primary antibody served as a negative control. After two additional PBS rinses and a brief dip in distilled water, coverslips were mounted on Superfrost glass slides with ProLong Glass Antifade Mountant containing NucBlue (Invitrogen). Fluorescent images were acquired using a Stellaris 5 laser-scanning confocal microscope (Leica Microsystems, Wetzlar, Germany) equipped with a 20× objective. Single-plan images were acquired using LAS X software (version 3.10.0.28982; Leica Microsystems), with identical laser power, detector gain, and pinhole settings applied to all experimental conditions.

### 4.3. Multiplex Cytokine Analysis

SIM-A9 microglial cells were seeded in a 24-well dish at 8 × 10^4^ cells and treated as described in Section 4.1. Cell culture supernatants were collected and stored at −80 °C until analysis. Cytokine concentrations in supernatants were measured using a magnetic bead-based multiplex assay (MILLIPLEX^®^ Mouse Cytokine/Chemokine Magnetic Bead Panel, Millipore Sigma, Darmstadt, Germany; MCYTOMAG-70K) according to the manufacturer’s instructions. Briefly, 25 μL of each sample was incubated overnight at 4 °C in the dark with 25 μL of an antibody-immobilized cytokine bead mixture per well in a 96-well MAG-PLATE, with shaking. After incubation, samples were washed twice with wash buffer and then incubated at room temperature in the dark with detection antibodies for 1 h, followed by incubation with streptavidin–phycoerythrin for 30 min. Samples were subsequently washed twice and resuspended in 150 μL of wash buffer before data acquisition on a Luminex MAGPIX^®^ instrument (MilliporeSigma, Burlington, MA, USA). Cytokine levels were analyzed using the Belysa® Immunoassay Curve Fitting software version 1.1.0 and were expressed in pg/mL. Data were graphed using GraphPad Prism version 10.2.3 (64-bit, 403).

### 4.4. Proteomic Sample Preparation

#### 4.4.1. Single-Pot Solid-Phase-Enhanced Sample Preparation—SP3

The Single-Pot Solid-Phase-enhanced Sample Preparation (SP3) method is a bead-based protein extraction method (which includes a mix of two sizes of paramagnetic carboxylate beads to capture a broad size range of peptides, is compatible with strong detergents, and includes streamlined binding and washing steps) described in detail elsewhere [20]. Briefly, cells were pelleted by centrifugation at 500× *g* for 5 min, and the supernatant was discarded. An ice-cold lysis buffer consisting of 1% sodium dodecyl sulfate (SDS) in PBS supplemented with protease inhibitors (Millipore Sigma, P8340) was added immediately before on-ice sonication using 10 s pulses. No sample required more than 30 s of sonication.

Protein concentration was determined using the Bradford assay. Volumes equivalent to 20 µg of protein were aliquoted and adjusted to a common volume with lysis buffer. Proteins were reduced with dithiothreitol (DTT) and subsequently alkylated with iodoacetamide (IAA) in 50 mM ammonium bicarbonate (AMBIC). A total of 200 µL of a 1:1 premixed suspension of modified carboxylate particles (GE Healthcare, Chicago, IL, USA, Cat#65152105050250 & 45152105050250) in 50 mM AMBIC was added to each sample. Protein binding to the beads was induced by adding an equal volume of 100% ethanol, followed by incubation for 15 min on a rotator. After removal of ethanol, bound proteins were washed three times with 80% ethanol to remove contaminants. Digestion was performed overnight at 37 °C with trypsin. The resulting digests, after bead removal, were frozen at −80 °C until acidified with formic acid and analyzed by mass spectrometry.

#### 4.4.2. Sample Preparation by Easy Extraction and Digestion—SPEED

Cells collected for sample preparation by easy extraction and digestion (SPEED) were pelleted by centrifugation as described for SP3 workflow and lysed using a detergent-free buffer consisting of PBS with protease inhibitors, followed by sonication as outlined above [21]. Protein concentration was determined using a Bradford assay. Volumes corresponding to 20 µg of protein were dried under vacuum, resuspended in pure trifluoroacetic acid (TFA), and incubated at room temperature for 20 min. The solution was neutralized with 2 M Tris base before adding 400 mM 2-chloroacetamide (CAA) and 100 mM Tris (2-hydroxyethyl) phosphine hydrochloride (TCEP). Reduction and alkylation were performed simultaneously at 95 °C for 5 min, and the reaction was quenched by adding a large volume of deionized water. Digestion with trypsin was carried out overnight at 37 °C. Peptide digests were desalted using solid-phase extraction (SPE) with C18 columns. Columns were washed with 50% methanol and equilibrated with 0.5% TFA in 5% acetonitrile (ACN) prior to sample loading. After loading, columns were centrifuged at 17,000× *g* for 1 min and washed with 0.5% TFA in 5% ACN. Peptides were eluted in 70% ACN, dried under vacuum, and resuspended in 0.1% formic acid (FA) for mass spectrometric analysis.

#### 4.4.3. Gel Enhanced Liquid Chromatography—GeLC

Approximately 20 µg of lysate prepared for SP3 was separated by sodium dodecyl sulfate polyacrylamide gel electrophoresis (SDS-PAGE) under reducing conditions. Briefly, Laemmli buffer (BioRad #1610747, Hercules, CA, USA) containing β-mercaptoethanol (Sigma-Aldrich, St. Louis, MO, USA) was added to the samples, which were then incubated for 5 min at 95 °C and loaded onto a 4–20% precast gel. Electrophoresis was carried out using 1× Tris/Glycine/SDS running buffer (BioRad #1610071) for 25 min at 300 V. The gel was washed three times with ddH_2_O before staining with GelCode Blue Stain Reagent (Thermo Scientific #24590, Waltham, CA, USA) for 1 h at room temperature with gentle shaking. The stain was then replaced with ddH_2_O for 1 h prior to imaging and excision. Images of the gels and the cutting scheme are available in Supplementary Figure S4.

Gels lanes were cut into 34 pieces using a 2 mm × 9 mm cutting grid (Gel Company, San Francisco, CA, USA, # MEE2-9-34), ensuring that the intensely stained ~150 kDa band was positioned in the sixth segment from the top of the gel, and that the bottom of the gel aligned with the bottom row of the grid. Every three adjacent gel pieces, starting from the top, were pooled to create bands 1–10; band 11 consisted of the last four pieces produced by the cutter grid. Gel bands were destained with 25 mM AMBIC containing 10% acetonitrile (ACN) under gentle shaking, replacing the solution as needed until no residual blue colour remained. Bands were then dehydrated in 90% ACN prior to reduction with 10 mM dithiothreitol in 50 mM AMBIC for 30 min. After removing the DDT solution, proteins were alkylated with 55 mM iodoacetamide for 60 min. Gel bands were again dehydrated in pure ACN before addition of trypsin in 25 mM AMBIC for overnight digestion at 37 °C. Peptide-containing supernatants were carefully removed to avoid disturbing the gel pieces, frozen, acidified with FA, and transferred to a siliconized 96-well “V-bottom” plate (Thermo Scientific TS-42800) for mass spectrometric analysis. Each gel band from the GeLC separation was run as an independent LC-MS/MS injection. Thus, for each gel lane (biological replicate) processed by GeLC, 11 raw files were produced. For data analysis in FragPipe, each gel band was processed independently and results combined later.

### 4.5. Mass Spectrometry Analysis

Samples were analyzed by label-free mass spectrometry-based quantification using data-dependent acquisition. For SP3 and SPEED acidified digests, ~0.1–0.2 µg of sample was loaded, and for Gel-LC band digests, 1/6th of each digest solution was loaded onto a reverse-phase UltiMate™ 3000 RSLD-nano system with Dionex ProFlow Meter (ThermoFisher Scientific, San Jose, CA, USA, Dionex). Briefly, 10 µL of peptide solution was concentrated on a PEPMAP NEO C18 5 µm trap (300 µm × 5 mm, Thermo Scientific) and then subsequently separated on a nanoAcquity UPLC M-Class 1.7 um BEH C18 column (100 µm × 100 mm), 130 Å pore size (Waters, Milford, MA, USA), using a flow rate of 500 nL/min with a 72 -min step-wise gradient of 1% to 6% Solvent B (Solvent A: 0.1% formic acid;, Solvent B: 100% acetonitrile (ACN)/0.1% formic acid) for 4 min, followed by a 48 min ramp to 25% Solvent B, a 9 min ramp to 40% Solvent B, another 3 min ramp to 85% Solvent B, and an 8 min equilibration at 1% Solvent B. Blanks with a 30 min gradient were run between samples to clean the system and reduce carry-over between runs. A full MS scan was acquired in the Orbitrap between 350 and 1800 *m*/*z* in profile mode at 60,000 resolution and was followed by a data-dependent MS/MS scan in the ion trap (IT) after higher-energy collisional dissociation (HCD) activation. Ions were excluded after a repeat count of 1 for a duration of 60 s. All data were recorded with Xcalibur software version 4.4.16.14 (build 6 February 2020) (ThermoFisher Scientific).

### 4.6. Data Processing

Raw data files from the mass spectrometer were processed using the FragPipe program (version 22.0; MSFragger 4.1; IonQuant 1.10.27; diaTracer 1.1.5; Philosopher 5.1.1). Briefly, files were loaded and assigned to experimental groups, and a database search was performed using the UniProt Mouse database (downloaded 12 August 2024) with common contaminants added and reverse-sequence decoys added for false-discovery rate calculation. Skyline (64-bit) 25.1.0.237 (519d29babc) was used for MS data validation and plotting. For STRING Protein–protein interaction map, Cytoscape version 3.10.3 was used with the following settings: Species = Mus musculus 2025-SEP-08; Network type = full STRING network; Confidence (score) cutoff > 0.80; Maximum additional interactors = 0.

### 4.7. Bioinformatics

Differential protein expression was assessed for all samples using the FragPipe-Analyst online tool with MaxLFQ Intensity data [35]. Variance-stabilizing normalization was applied, followed by Perseus-type imputation. *p*-values obtained from statistical analysis were corrected using Local and tail area-based methods for GeLC and Benjamini–Hochberg for SP3 and SPEED samples. Report files were subsequently downloaded for data exploration and visualization. Corrected *p*-values were considered significant at *p* < 0.05 unless otherwise specified. Groups of differentially expressed proteins were analyzed by G:Profiler, and results from KEGG and Gene Ontology (GO) enrichment were downloaded for visualization in R [36]. Qualitative assessment of protein physical and biochemical characteristics was performed using a combination of in-house Perl scripts, available data sources, and Microsoft Excel. Graphical representations were generated using custom R scripts (version 4.4.1 using RStudio 2024.04.2 + 764) or GraphPad Prism (version 10.6.1 (64-bit, 892)).

## Data Availability

The original contributions presented in this study are included in the article/Supplementary Materials. Further inquiries can be directed to the corresponding authors.

## Supplementary Materials

The following supporting information can be downloaded at: https://www.mdpi.com/article/10.3390/ijms27020689/s1.

## Abbreviations

The following abbreviations are used in this manuscript:

ACN	Acetonitrile
AMBIC	Ammonium Bicarbonate
ATP	Adenosine Triphosphate
CAA	2-chloroacetamide
DAMP	Damage-associated Molecular Pattern
DTT	Dithiothreitol
ESI	Electrospray Ionization
GeLC	Gel Enhanced Liquid Chromatography
HSP	Heat Shock Protein
IAA	Iodoacetamide
KEGG	Kyoto Encyclopedia of Genes and Genomes
LC-MS/MS	Liquid Chromatography Tandem Mass Spectrometry
LPS	Lipopolysaccharide
mtDNA	Mitochondrial Deoxyribonucleic Acid
NG	Nigericin
PAMP	Pathogen-associated Molecular Pattern
PBS	Phosphate-Buffered Saline
PRR	Pattern Recognition Receptor
SDS-PAGE	Sodium Dodecyl Sulfate Polyacrylamide Gel Electrophoresis
SF	Serum-free
SP3	Single-Pot, Solid-Phase-Enhanced Sample Preparation
SPE	Solid Phase Extraction
SPEED	Sample Preparation by Easy Extraction and Digestion
TCEP	Tris (2-hydroxymethyl) phosphine hydrochloride
