# Supplementary material for: Characterization of the Proteomic Response in SIM-A9 Murine Microglia Following Canonical NLRP3 Inflammasome Activation
Source: Int J Mol Sci. 2026 Jan 9;27(2):689. doi: 10.3390/ijms27020689 (PMC12840609; doi:10.3390/ijms27020689)
Supplement: Supplementary file 1 [file ijms-27-00689-s001.zip › ijms-4047182-Supplementary_Figures.pptx]

## Slide 1
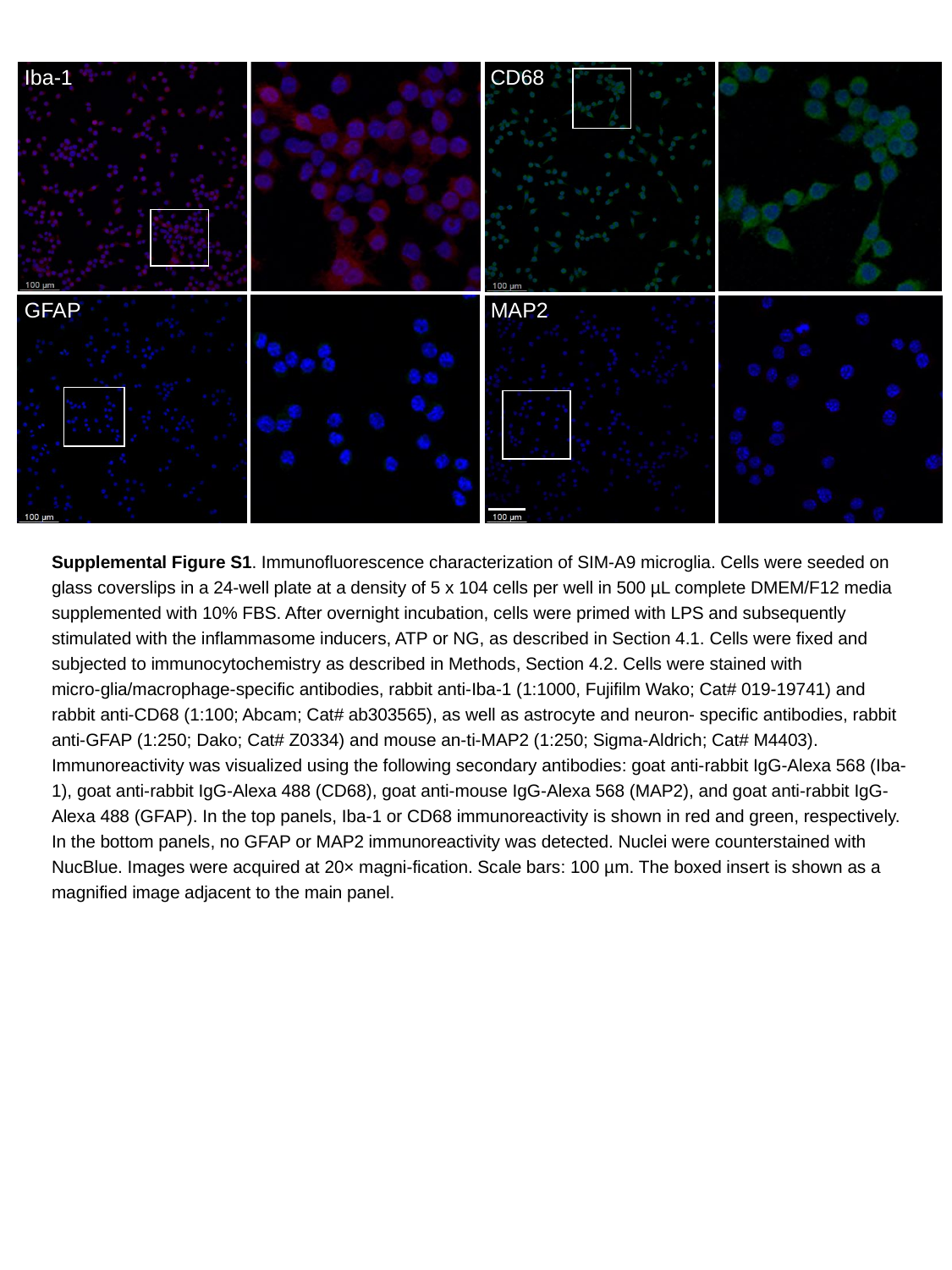

Iba-1
CD68
GFAP
MAP2
Supplemental Figure S1. Immunofluorescence characterization of SIM-A9 microglia. Cells were seeded on glass coverslips in a 24-well plate at a density of 5 x 10^4^ cells per well in 500 µL complete DMEM/F12 media supplemented with 10% FBS. After overnight incubation, cells were primed with LPS and subsequently stimulated with the inflammasome inducers, ATP or NG, as described in Section 4.1. Cells were fixed and subjected to immunocytochemistry as described in Methods, Section 4.2. Cells were stained with micro-glia/macrophage-specific antibodies, rabbit anti-Iba-1 (1:1000, Fujifilm Wako; Cat# 019-19741) and rabbit anti-CD68 (1:100; Abcam; Cat# ab303565), as well as astrocyte and neuron- specific antibodies, rabbit anti-GFAP (1:250; Dako; Cat# Z0334) and mouse an-ti-MAP2 (1:250; Sigma-Aldrich; Cat# M4403). Immunoreactivity was visualized using the following secondary antibodies: goat anti-rabbit IgG-Alexa 568 (Iba-1), goat anti-rabbit IgG-Alexa 488 (CD68), goat anti-mouse IgG-Alexa 568 (MAP2), and goat anti-rabbit IgG-Alexa 488 (GFAP). In the top panels, Iba-1 or CD68 immunoreactivity is shown in red and green, respectively. In the bottom panels, no GFAP or MAP2 immunoreactivity was detected. Nuclei were counterstained with NucBlue. Images were acquired at 20× magni-fication. Scale bars: 100 µm. The boxed insert is shown as a magnified image adjacent to the main panel.

## Slide 2
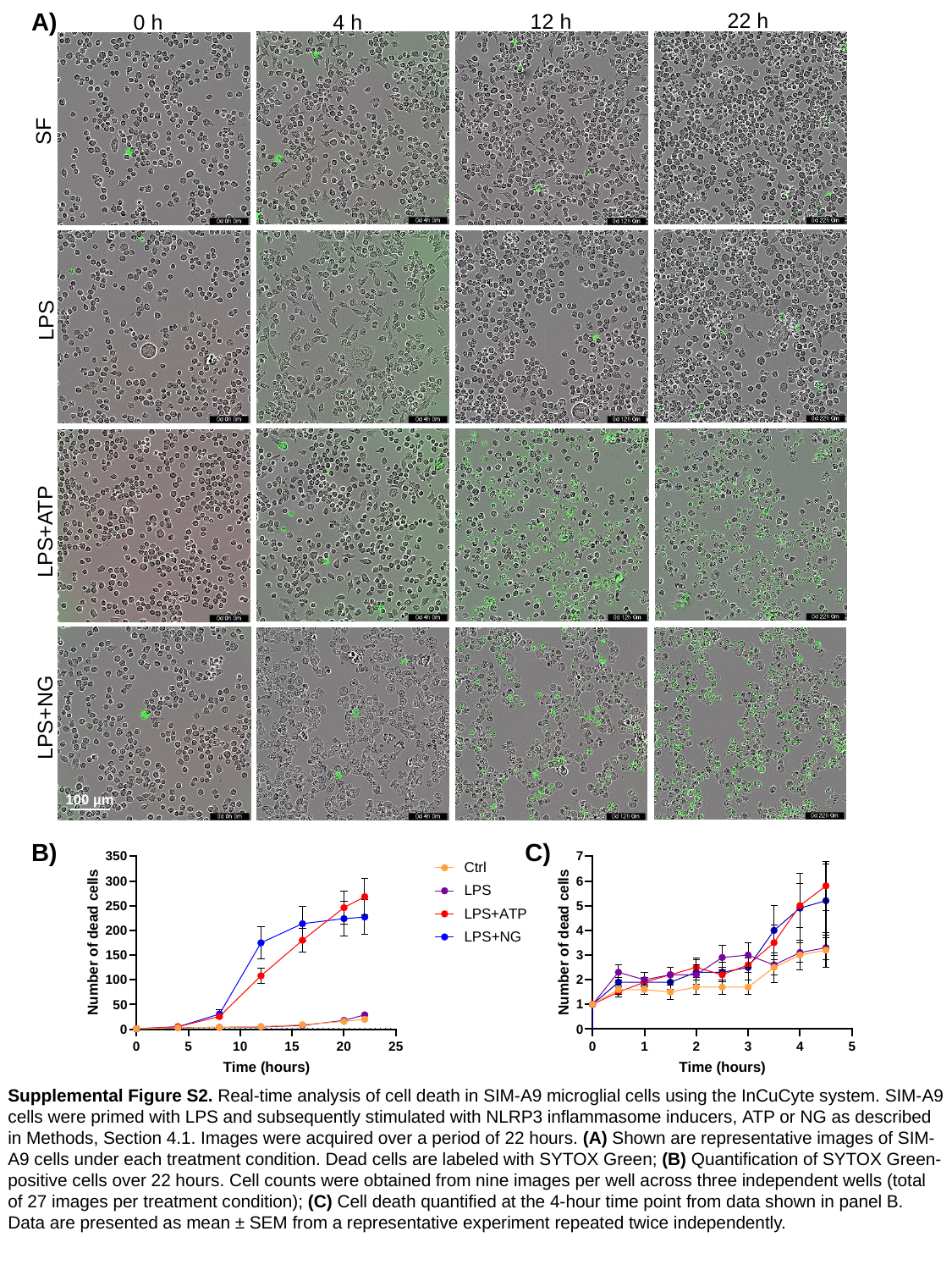

A)
22 h
12 h
4 h
0 h
SF
LPS
LPS+ATP
100 µm
LPS+NG
B)
C)
Supplemental Figure S2. Real-time analysis of cell death in SIM-A9 microglial cells using the InCuCyte system. SIM-A9 cells were primed with LPS and subsequently stimulated with NLRP3 inflammasome inducers, ATP or NG as described in Methods, Section 4.1. Images were acquired over a period of 22 hours. (A) Shown are representative images of SIM-A9 cells under each treatment condition. Dead cells are labeled with SYTOX Green; (B) Quantification of SYTOX Green-positive cells over 22 hours. Cell counts were obtained from nine images per well across three independent wells (total of 27 images per treatment condition); (C) Cell death quantified at the 4-hour time point from data shown in panel B. Data are presented as mean ± SEM from a representative experiment repeated twice independently.

## Slide 3
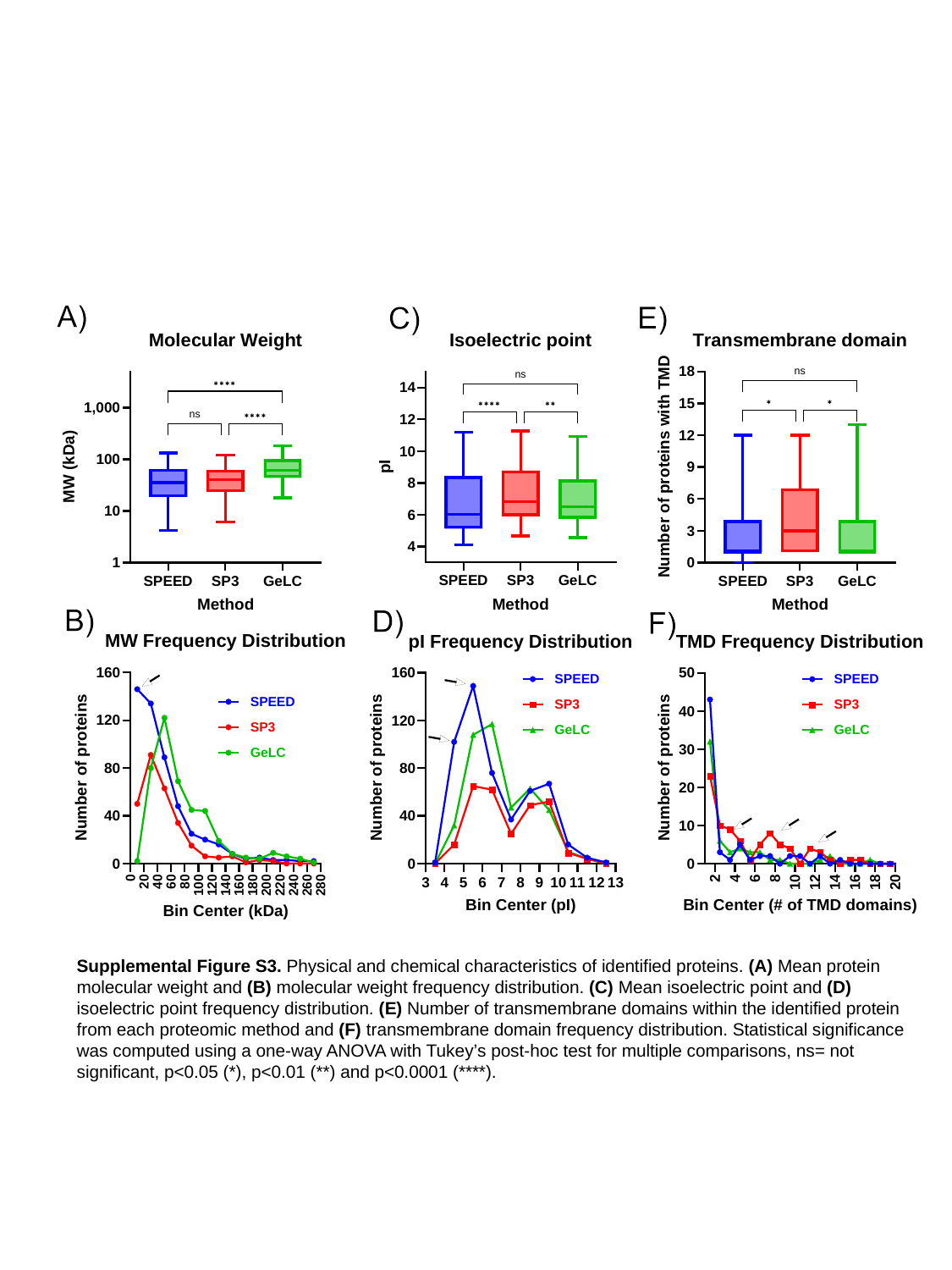

Supplemental Figure S3. Physical and chemical characteristics of identified proteins. (A) Mean protein molecular weight and (B) molecular weight frequency distribution. (C) Mean isoelectric point and (D) isoelectric point frequency distribution. (E) Number of transmembrane domains within the identified protein from each proteomic method and (F) transmembrane domain frequency distribution. Statistical significance was computed using a one-way ANOVA with Tukey’s post-hoc test for multiple comparisons, ns= not significant, p<0.05 (*), p<0.01 (**) and p<0.0001 (****).

## Slide 4
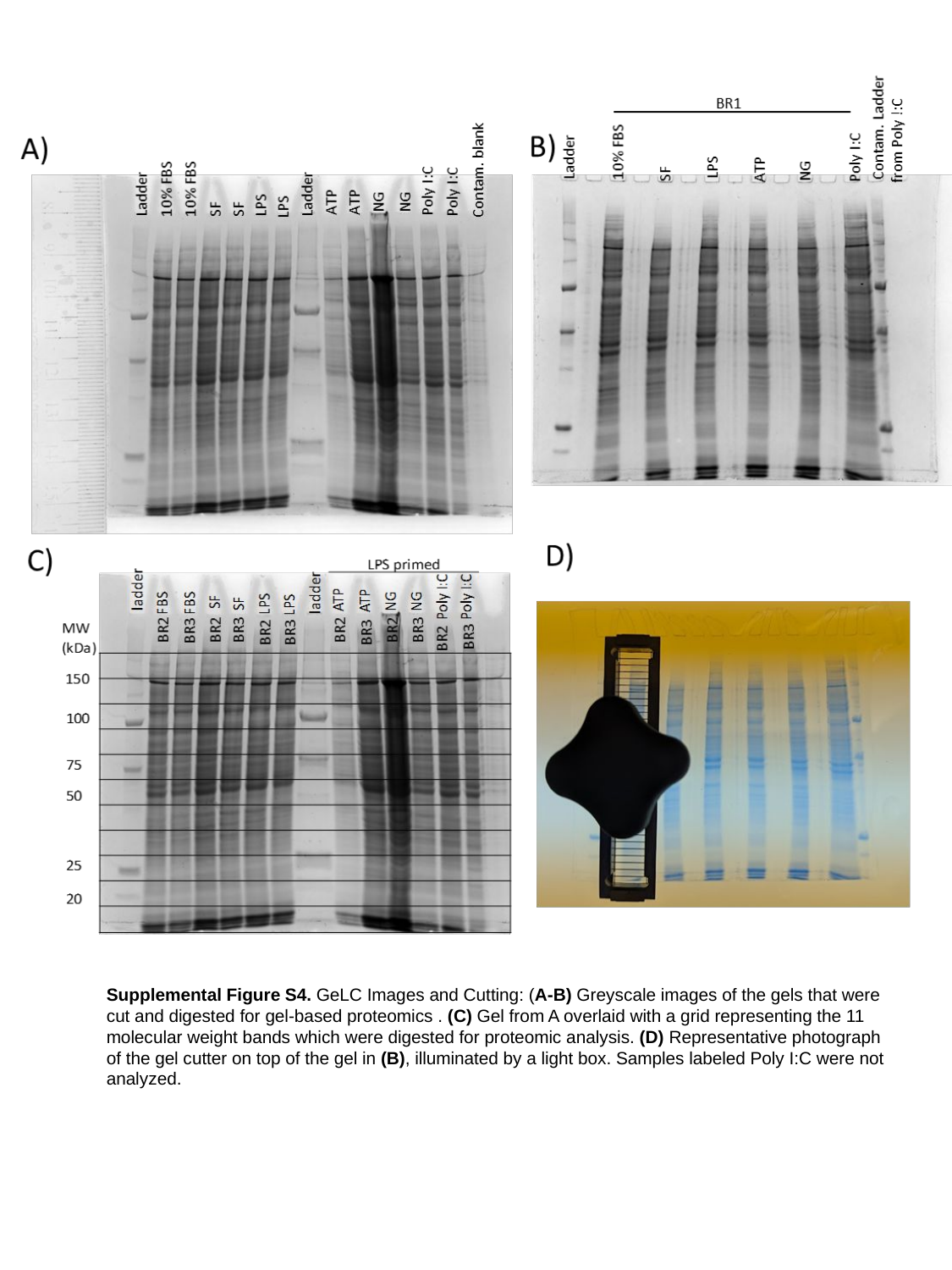

Supplemental Figure S4. GeLC Images and Cutting: (A-B) Greyscale images of the gels that were cut and digested for gel-based proteomics . (C) Gel from A overlaid with a grid representing the 11 molecular weight bands which were digested for proteomic analysis. (D) Representative photograph of the gel cutter on top of the gel in (B), illuminated by a light box. Samples labeled Poly I:C were not analyzed.
