# Supplementary material for: Characterization of the Proteomic Response in SIM-A9 Murine Microglia Following Canonical NLRP3 Inflammasome Activation
Source: Int J Mol Sci. 2026 Jan 9;27(2):689. doi: 10.3390/ijms27020689 (PMC12840609; doi:10.3390/ijms27020689)
Supplement: Supplementary file 1 [file ijms-27-00689-s001.zip › ijms-4047182-Supplementary_Methods.pdf]

Supplementary Methods

Assessment of cell death

Cell death was assessed using the SYTOX™ Green nucleic acid stain (Invitrogen, Cat#S7020) in combination with real-time imaging on the SX5 IncuCyte® Live-Cell Analysis System (Sartorius). SIM-A9 microglial cells were seeded in a 24-well dish at 8 x 10^4 cells per well and treated under the experimental conditions as described in the Methods Section 4.1. SYTOX™ Green was added to the culture medium at a final concentration of 1.25 μM to selectively label cells with compromised plasma membranes. Plates were placed overnight in the IncuCyte® instrument maintained at 37 °C with 5% CO_2 for real-time cell death assay. Nine images per well were acquired every 30 min using phase contrast and green fluorescence channels (excitation: 440–480 nm; emission: 504–544 nm). The number of SYTOX™ Green-positive cells and total cell area were automatically quantified using IncuCyte® analysis software. Data were analyzed and graphed using GraphPad Prism version 10.2.3 (64-bit, 403). Cell death was expressed as the number of fluorescent (dead) cells normalized to number of dead cells at time 0.
